# Prior Sensitivity Analysis in a Semi-Parametric Integer-Valued Time Series Model

**DOI:** 10.3390/e22010069

**Published:** 2020-01-06

**Authors:** Helton Graziadei, Antonio Lijoi, Hedibert F. Lopes, Paulo C. Marques F., Igor Prünster

**Affiliations:** 1 Instituto de Matemática e Estatística, Universidade de São Paulo, São Paulo 05508-090, Brazil; 2 Department of Decision Sciences and BIDSA, Bocconi University, via Röntgen 1, 20136 Milano, Italy; antonio.lijoi@unibocconi.it (A.L.); igor.pruenster@unibocconi.it (I.P.); 3 Insper Institute of Education and Research, Rua Quatá 300, São Paulo 04546-042, Brazil; HedibertFL@insper.edu.br (H.F.L.); PauloCMF1@insper.edu.br (P.C.M.F.)

**Keywords:** time series of counts, Bayesian hierarchical modeling, Bayesian nonparametrics, Pitman–Yor process, prior sensitivity, clustering, Bayesian forecasting

## Abstract

We examine issues of prior sensitivity in a semi-parametric hierarchical extension of the INAR(*p*) model with innovation rates clustered according to a Pitman–Yor process placed at the top of the model hierarchy. Our main finding is a graphical criterion that guides the specification of the hyperparameters of the Pitman–Yor process base measure. We show how the discount and concentration parameters interact with the chosen base measure to yield a gain in terms of the robustness of the inferential results. The forecasting performance of the model is exemplified in the analysis of a time series of worldwide earthquake events, for which the new model outperforms the original INAR(*p*) model.

## 1. Introduction

Integer-valued time series are relevant to many fields of knowledge, ranging from finance and econometrics to ecology and meteorology. An extensive number of models for this kind of data has been proposed since the introduction of the INAR(1) model in the pioneering works of McKenzie [1] and Al-Osh and Alzaid [2] (see also the book by Weiss [3]). A higher-order INAR(*p*) model was considered in the work of Du and Li [4].

In this paper, we generalize the Bayesian version of the INAR(*p*) model studied by Neal and Kypraios [5]. In our model, the innovation rates are allowed to vary through time, with the distribution of the innovation rates being modeled hierarchically by means of a Pitman–Yor process [6]. In this way, we account for potential heterogeneity in the innovation rates as the process evolves through time, and this feature is automatically incorporated in the Bayesian forecasting capabilities of the model.

The semi-parametric form of the model demands a robustness analysis of our inferential conclusions as we vary the hyperparameters of the Pitman–Yor process. We investigate this prior sensitivity issue carefully and find ways to control the hyperparameters in order to achieve robust results.

This paper is organized as follows. In Section 2, we construct a generalized INAR(*p*) model with variable innovation rates. The likelihood function of the generalized model is derived and a data augmentation scheme is developed, which gives a specification of the model in terms of conditional distributions. This data augmented representation of the model enables the derivation in Section 4 of full conditional distributions in simple analytical form, which are essential for the stochastic simulations in Section 5. Section 3 recollects the main properties of the Pitman–Yor process which are used to define the PY-INAR(*p*) model in Section 4, including its clustering properties. In building the PY-INAR(*p*), we propose a form for the prior distribution of the thinning parameters vector which improves on the choice made for the Bayesian INAR(*p*) model studied in [5]. In Section 5, we investigate the robustness of the inference with respect to changes in the Pitman–Yor process hyperparameters. Using the full conditional distributions of the innovation rates derived in Section 4, we inspect the behavior of the model as we concentrate or spread the mass of the Pitman–Yor base measure. This leads us to a graphical criterion that identifies an elbow in the posterior expectation of the number of clusters as we vary the hyperparameters of the base measure. Once we have control over the base measure, we study its interaction with the concentration and discount hyperparameters, showing how to make choices that yield robust results. In the course of this development, we use geometrical tools to inspect the clustering of the innovation rates produced by the model. Section 6 puts the graphical criterion to work for simulated data. In Section 7, using a time series of worldwide earthquake events, we finish the paper comparing the forecasting performance of the PY-INAR(*p*) model against the original INAR(*p*) model, with favorable results.

## 2. A Generalization of the INAR(*p*) Model

We begin by generalizing the original INAR(*p*) model of Du and Li [4] as follows.

Let ${\left\{Y_{t}\right\}}_{t\geq 1}$ be an integer-valued time series, and, for some integer p≥1, let the *innovations*${\left\{Z_{t}\right\}}_{t\geq p+1}$, given positive parameters ${\left\{\lambda_{t}\right\}}_{t\geq p+1}$, be a sequence of conditionally independent $\mathrm{Poisson}(\lambda_{t})$ random variables. For a given vector of parameters $\alpha =\left(\alpha_{1},\cdots ,\alpha_{p}\right)\in {\left[0,1\right]}^{p}$, let $F_{i}=\left\{B_{ij}\left(t\right):j\geq 0,t\geq 2\right\}$ be a family of conditionally independent and identically distributed $\mathrm{Bernoulli}(\alpha_{i})$ random variables. For i≠k, suppose that $F_{i}$ and $F_{k}$ are conditionally independent, given α. Furthermore, assume that the innovations ${\left\{Z_{t}\right\}}_{t\geq p+1}$ and the families $F_{1},\cdots ,F_{p}$ are conditionally independent, given α and λ. The generalized INAR(*p*) model is defined by the functional relation

$$Y_{t}=\alpha_{1}\circ Y_{t-1}+\cdots +\alpha_{p}\circ Y_{t-p}+Z_{t},$$

for t≥p+1, in which ∘ denotes the binomial thinning operator, defined by $\alpha_{i}\circ Y_{t-i}=\sum_{j=1}^{Y_{t-i}}B_{ij}\left(t\right)$, if $Y_{t-i}>0$, and $\alpha_{i}\circ Y_{t-i}=0$, if $Y_{t-i}=0$. In the homogeneous case, when all the $\lambda_{t}$’s are assumed to be equal, we recover the original INAR(*p*) model.

When p=1, this model can be interpreted as specifying a birth-and-death process, in which, at epoch *t*, the number of cases $Y_{t}$ is equal to the new cases $Z_{t}$ plus the cases that survived from the previous epoch; the role of the binomial thinning operator being to remove a random number of the $Y_{t-1}$ cases present at the previous epoch t−1 (see [7] for an interpretation of the order *p* case as a birth-and-death process with immigration).

Let $y=(y_{1},\cdots ,y_{T})$ denote the values of an observed time series. For simplicity, we assume that $Y_{1}=y_{1},\ldots ,Y_{p}=y_{p}$ with probability one. The joint distribution of $Y_{1},\cdots ,Y_{T}$, given parameters α and $\lambda =(\lambda_{p+1},\cdots ,\lambda_{T})$, can be factored as

$$\mathrm{Pr}\left\{Y_{1}=y_{1},\cdots ,Y_{T}=y_{T}|\alpha ,\lambda \right\}=\prod_{t=p+1}^{T}\mathrm{Pr}\left\{Y_{t}=y_{t}|Y_{t-1}=y_{t-1},\ldots ,Y_{t-p}=y_{t-p},\alpha ,\lambda_{t}\right\}.$$

Since, with probability one, $\alpha_{i}\circ Y_{t-i}\leq Y_{t-i}$ and $Z_{t}\geq 0$, the likelihood function of the generalized INAR(*p*) model is given by

Ly(α,λ)=∏t=p+1T∑m1,t=0min{yt,yt−1}⋯∑mp,t=0min{yt−∑j=1p−1mj,t,yt−p}∏i=1pyt−imi,tαimi,t(1−αi)yt−i−mi,t×e−λtλtyt−∑j=1pmj,t(yt−∑j=1pmj,t)!.

For some epoch *t* and i=1,⋯,p, suppose that we could observe the values of the latent *maturations*$M_{i,t}$. Postulate that $M_{i,t}|Y_{t-i}=y_{t-i},\alpha_{i}∼\mathrm{Binomial}\left(y_{t-i},\alpha_{i}\right)$, so that the conditional probability function of $M_{i,t}$ is given by

p(mi,t∣yt−i,αi)=Pr{Mi,t=mi,t∣Yt−i=yt−i,αi}=yt−imi,tαimi,t(1−αi)yt−i−mi,tI{0,⋯,yt−i}(mi,t).

Furthermore, suppose that

$$\begin{array}{cc} p(y_{t}|m_{1,t},\ldots ,m_{p,t},\lambda_{t}) & =\mathrm{Pr}\{Y_{t}=y_{t}|M_{1,t}=m_{1,t},\cdots ,M_{p,t}=m_{p,t},\lambda_{t}\}\\ & =\frac{e^{-\lambda_{t}}\lambda_{t}^{y_{t}-\sum_{j=1}^{p}m_{j,t}}}{(y_{t}-\sum_{j=1}^{p}m_{j,t})!}\;I_{\left\{\sum_{j=1}^{p}m_{j,t},\;\sum_{j=1}^{p}m_{j,t}+1,\;\cdots \right\}}\left(y_{t}\right). \end{array}$$

Using the law of total probability and the product rule, we have that

$$\begin{array}{cc} p(y_{t}|y_{t-1},\ldots ,y_{t-p},\alpha ,\lambda_{t}) & =\sum_{m_{1,t}=0}^{y_{t-1}}\cdots \sum_{m_{p,t}=0}^{y_{t-p}}p\left(y_{t},m_{1,t},\ldots ,m_{p,t}|y_{t-1},\ldots ,y_{t-p},\alpha ,\lambda_{t}\right)\\ \; & =\sum_{m_{1,t}=0}^{y_{t-1}}\cdots \sum_{m_{p,t}=0}^{y_{t-p}}p\left(y_{t}|m_{1,t},\ldots ,m_{p,t},\lambda_{t}\right)\times \prod_{i=1}^{p}p\left(m_{i,t}|y_{t-i},\alpha_{i}\right). \end{array}$$

Since

$$\begin{array}{cc} I_{\left\{\sum_{j=1}^{p}m_{j,t},\;\sum_{j=1}^{p}m_{j,t}+1,\;\cdots \right\}}\left(y_{t}\right) & =I_{\left\{0,\;\cdots \;,\;y_{t}\right\}}\;\left(\sum_{j=1}^{p}m_{j,t}\right)\\ \; & =I_{\left\{0,\;\cdots \;,\;y_{t}\right\}}\left(m_{1,t}\right)\times \cdots \times I_{\left\{0,\;\cdots \;,\;y_{t}-\sum_{j=1}^{p-1}m_{j,t}\right\}}\left(m_{p,t}\right) \end{array}$$

and

$$I_{\left\{\sum_{j=1}^{p}m_{j,t},\;\sum_{j=1}^{p}m_{j,t}+1,\;\cdots \right\}}\left(y_{t}\right)\times I_{\left\{0,\;\cdots \;,\;y_{t-i}\right\}}\left(m_{i,t}\right)=I_{\left\{0,\;1,\;\cdots \;,\;\min\left\{y_{t}-\sum_{j\neq i}m_{j,t},\;y_{t-i}\right\}\right\}}\left(m_{i,t}\right),$$

we recover the original likelihood of the generalized INAR(*p*), showing that the introduction of the latent maturations $M_{i,t}$ with the specified distributions is a valid data augmentation scheme (see [8,9] for a general discussion of data augmentation techniques).

In the next section, we review the needed definitions and properties of the Pitman–Yor process.

## 3. Pitman–Yor Process

Let the random probability measure $G∼\mathrm{DP}(\tau ,G_{0})$ be a Dirichlet process [10,11,12] with concentration parameter τ and base measure $G_{0}$. If the random variables $X_{1},\cdots ,X_{n}$, given G=G, are conditionally independent and identically distributed as *G*, then it follows that

$$\mathrm{Pr}\left\{X_{n+1}\in B|X_{1}=x_{1},\cdots ,X_{n}=x_{n}\right\}=\frac{\tau }{\tau +n}\;G_{0}\left(B\right)+\frac{1}{\tau +n}\sum_{i=1}^{n}I_{B}\left(x_{i}\right),$$

for every Borel set *B*. If we imagine the sequential generation of the $X_{i}$’s, for i=1,⋯,n, the former expression shows that a value is generated anew from $G_{0}$ with probability proportional to τ, or we repeat one the previously generated values with probability proportional to its multiplicity. Therefore, almost surely, realizations of a Dirichlet process are discrete probability measures, perhaps with denumerable infinite support, depending on the nature of $G_{0}$. Also, this data-generating process, known as the Pólya–Blackwell–MacQueen urn, implies that the $X_{i}$’s are “softly clustered”, in the sense that in one realization of the process the elements of a subset of the $X_{i}$’s may have exactly the same value.

The Pitman–Yor process [6] is a generalization of the Dirichlet process which results in a model with added flexibility. Essentially, the Pitman–Yor process modifies the expression of the probability associated with the Pólya-Blackwell-MacQueen urn introducing a new parameter so that the posterior predictive probability becomes

$$\mathrm{Pr}\left\{X_{n+1}\in B|X_{1}=x_{1},\cdots ,X_{n}=x_{n}\right\}=\frac{\tau +k\sigma }{\tau +n}\;G_{0}\left(B\right)+\frac{1}{\tau +n}\sum_{i=1}^{n}\left(1-\frac{\sigma }{n_{i}}\right)I_{B}\left(x_{i}\right),$$

in which 0≤σ<1 is the discount parameter, τ>−σ, *k* is the number of distinct elements in $\left\{X_{1},\cdots ,X_{n}\right\}$, and $n_{i}$ is the number of elements in $\left\{X_{1},\ldots ,X_{n}\right\}$ which are equal to $X_{i}$, for i=1,⋯,n. It is well known that $E\left[G\left(B\right)\right]=G_{0}\left(B\right)$ and

$$\mathrm{Var}\left[G\left(B\right)\right]=\left(\frac{1-\sigma }{\tau +1}\right)G_{0}\left(B\right)\left(1-G_{0}\left(B\right)\right),$$

for every Borel set *B*. Hence, G is centered on the base probability measure $G_{0}$, while τ and σ control the concentration of G around $G_{0}$. We use the notation $G∼\mathrm{PY}(\tau ,\sigma ,G_{0})$. When σ=0, we recover the Dirichlet process as a special case. The PY process is also defined for σ<0 and τ=|σ|m, for some positive integer *m*. For our purposes, it is enough to consider the case of non-negative σ.

Pitman [6] derived the distribution of the number of clusters *K* (the number of distinct $X_{i}$’s), conditionally on both the concentration parameter τ and the discount parameter σ, as

$$\mathrm{Pr}\left\{K=k|\tau ,\sigma \right\}=\frac{\prod_{i=1}^{k-1}\left(\tau +i\sigma \right)}{\sigma^{k}\times {\left(\tau +1\right)}_{n-1}}\times C\left(n,k;\sigma \right),$$

in which ${\left(x\right)}_{n}=\Gamma \left(x+n\right)/\Gamma \left(x\right)$ is the rising factorial and C(n,k;σ) is the generalized factorial coefficient [13].

In the next section, we use a Pitman–Yor process to model the distribution of the innovation rates in the generalized INAR(*p*) model.

## 4. PY-INAR(*p*) Model

The PY-INAR(*p*) model is as a hierarchical extension of the generalized INAR(*p*) model defined in Section 2. Given a random measure $G∼\mathrm{PY}(\tau ,\sigma ,G_{0})$, in which $G_{0}$ is a $\mathrm{Gamma}(a_{0},b_{0})$ distribution, let the innovation rates $\lambda_{p+1},\cdots ,\lambda_{T}$ be conditionally independent and identically distributed with distribution $\mathrm{Pr}\left\{\lambda_{t}\in B|G=G\right\}=G\left(B\right)$.

To complete the PY-INAR(*p*) model, we need to specify the form of the prior distribution for the vector of thinning parameters $\alpha =(\alpha_{1},\cdots ,\alpha_{p})$. By comparison with standard results from the theory of the AR(*p*) model [14], Du and Li [4] found that in the INAR(*p*) model the constraint $\sum_{i=1}^{p}\alpha_{i}<1$ must be fulfilled to guarantee the non-explosiveness of the process. In their Bayesian analysis of the INAR(*p*) model, Neal and Kypraios [5] considered independent beta distributions for the $\alpha_{i}$’s. Unfortunately, this choice is problematic. For example, in the particular case when the $\alpha_{i}$’s have independent uniform distributions, it is possible to show that $\mathrm{Pr}\{\sum_{i=1}^{p}\alpha_{i}<1\}=1/p!$, implying that we would be concentrating most of the prior mass on the explosive region even for moderate values of the model order *p*. We circumvent this problem using a prior distribution for α that places all of its mass on the nonexplosive region and still allows us to derive the full conditional distributions of the $\alpha_{i}$’s in simple closed form. Specifically, we take the prior distribution of α to be a Dirichlet distribution with hyperparameters $\left(a_{1},\cdots ,a_{p};a_{p+1}\right)$, and corresponding density

$$\pi \left(\alpha \right)=\frac{\Gamma \left(\sum_{i=1}^{p+1}a_{i}\right)}{\prod_{i=1}^{p+1}\Gamma \left(a_{i}\right)}\prod_{i=1}^{p+1}\alpha_{i}^{a_{i}-1},$$

in which $a_{i}>0$, for i=1,⋯,p+1, and $\alpha_{p+1}=1-\sum_{i=1}^{p}\alpha_{i}$.

Let $m=\{m_{i,t}$: i=1,…,p, t=p+1,…,T} denote the set of all maturations, and let $\mu_{G}$ be the distribution of G. Our strategy to derive the full conditionals distributions of the model parameters and latent variables is to consider the marginal distribution

$$\begin{array}{cc} p(y,m,\alpha ,\lambda ) & =\int p\left(y,m,\alpha ,\lambda |G\right)\;d\mu_{G}\left(G\right)\\ \; & =\left\{\prod_{t=p+1}^{T}p\left(y_{t}|m_{1,t},\ldots ,m_{p,t},\lambda_{t}\right)\;\prod_{i=1}^{p}p\left(m_{i,t}|y_{t-i},\alpha_{i}\right)\right\}\\ \; & \;\times \pi \left(\alpha \right)\times \int \prod_{t=p+1}^{T}p\left(\lambda_{t}|G\right)\;d\mu_{G}\left(G\right). \end{array}$$

From this expression, using the results in Section 3, the derivation of the full conditional distributions is straightforward. In the following expressions, the symbol ∝ denotes proportionality up to a suitable normalization factor, and the label “all others” designate the observed counts *y* and all the other latent variables and model parameters, with the exception of the one under consideration.

Let $\lambda_{\backslash t}$ denote the set $\left\{\lambda_{p+1},\cdots ,\lambda_{T}\right\}$ with the element $\lambda_{t}$ removed. Then, for t=p+1,⋯,T, we have

$$\lambda_{t}|\mathrm{all}\;\mathrm{others}∼w_{t}\times \mathrm{Gamma}\left(y_{t}-m_{t}+a_{0},b_{0}+1\right)+\sum_{r\neq t}\left(1-\frac{\sigma }{n_{r}}\right)\;\lambda_{r}^{y_{t}-m_{t}}e^{-\lambda_{r}}\delta_{\left\{\lambda_{r}\right\}},$$

in which the weight

$$w_{t}=\frac{\left(\tau +k_{\backslash t}\;\sigma \right)\times b_{0}^{a_{0}}\times \Gamma \left(y_{t}-m_{t}+a_{0}\right)}{\Gamma \left(a_{0}\right)\times {\left(b_{0}+1\right)}^{y_{t}-m_{t}+a_{0}}},$$

$n_{r}$ is the number of elements in $\lambda_{\backslash t}$ which are equal to $\lambda_{r}$, and $k_{\backslash t}$ is the number of distinct elements in $\lambda_{\backslash t}$. In this mixture, we suppressed the normalization constant that makes all weights add up to one.

Making the choice $a_{p+1}=1$, we have

$$\alpha_{i}|\mathrm{all}\;\mathrm{others}∼\mathrm{TBeta}\;\left(a_{i}+\sum_{t=p+1}^{T}m_{i,t},1+\sum_{t=p+1}^{T}\left(y_{t-i}-m_{i,t}\right),1-\sum_{j\neq i}\alpha_{j}\right),$$

for i=1,⋯,p, in which TBeta denotes the right truncated Beta distribution with support $\left(0,1-\sum_{j\neq i}^{p}\alpha_{j}\right)$.

For the latent maturations, we find

$$\begin{array}{cc} p(m_{i,t}|\mathrm{all}\;\mathrm{others}) & \propto \frac{1}{\left(m_{i,t}\right)!\left(y_{t}-\sum_{j=1}^{p}m_{j,t}\right)!\left(y_{t-i}-m_{i,t}\right)!}{\left(\frac{\alpha_{i}}{\lambda_{t}\left(1-\alpha_{i}\right)}\right)}^{m_{i,t}}\\ \; & \;\times I_{\left\{0,\;1,\;\cdots \;,\;\min\left\{y_{t}-\sum_{j\neq i}m_{j,t},\;y_{t-i}\right\}\right\}}\left(m_{i,t}\right). \end{array}$$

To explore the posterior distribution of the model, we build a Gibbs sampler [15] using these full conditional distributions. Escobar and West [16] showed, in a similar context, that we can improve mixing by resampling simultaneously the values of all $\lambda_{t}$’s inside the same cluster at the end of each iteration of the Gibbs sampler. Letting $\left(\lambda_{1}^{*},\cdots ,\lambda_{k}^{*}\right)$ be the *k* unique values among $\left(\lambda_{p+1},\cdots ,\lambda_{T}\right)$, define the number of occupants of cluster *j* by $\nu_{j}=\sum_{t=p+1}^{T}I_{\left\{\lambda_{j}^{*}\right\}}\left(\lambda_{t}\right)$, for j=1,…,k. It follows that

$$\lambda_{j}^{*}|\mathrm{all}\;\mathrm{others}∼\mathrm{Gamma}\;\left(a_{0}+\sum_{t=p+1}^{T}\left(y_{t}-\sum_{i=1}^{p}m_{i,t}\right)\cdot I_{\left\{\lambda_{j}^{*}\right\}}\left(\lambda_{t}\right),b_{0}+\nu_{j}\right).$$

for j=1,…,k. At the end of each iteration of the Gibbs sampler, we update the values of all $\lambda_{t}$’s inside each cluster by the corresponding $\lambda_{j}^{*}$ using this distribution.

## 5. Prior Sensitivity

As it is often the case for Bayesian models with nonparametric components, a choice of the prior parameters for the PY-INAR(*p*) model which yields robustness of the posterior distribution is nontrivial [17].

The first aspect to be considered is the fact that the base measure $G_{0}$ plays a crucial role in the determination of the posterior distribution of the number of clusters *K*. This can be seen directly by inspecting the form of the full conditional distributions derived in Section 4. Recalling that $G_{0}$ is a gamma distribution with mean $a_{0}/b_{0}$ and variance $a_{0}/b_{0}^{2}$, from the full conditional distribution of $\lambda_{t}$ one may note that the probability of generating, on each iteration of the Gibbs sampler, a value for $\lambda_{t}$ anew from $G_{0}$ is proportional to

$$\frac{\left(\tau +k_{\backslash t}\;\sigma \right)\times b_{0}^{a_{0}}\times \Gamma \left(y_{t}-m_{t}+a_{0}\right)}{\Gamma \left(a_{0}\right){\left(b_{0}+1\right)}^{y_{t}-m_{t}+a_{0}}}.$$

Therefore, supposing that all the other terms are fixed, if we concentrate the mass of $G_{0}$ around zero by making $b_{0}\to \infty$, this probability decreases to zero. This is not problematic, because it is hardly the case that we want to make such a drastic choice for $G_{0}$. The behavior in the other direction is more revealing, since taking $b_{0}↓0$, in order to spread the mass of $G_{0}$, also makes the limit of this probability to be zero. Due to this behavior, we need to establish a criterion to choose the hyperparameters of the base measure which avoids these extreme cases.

In our analysis, it is convenient to have a single hyperparameter regulating how the mass of $G_{0}$ is spread over its support. For a given $\lambda_{\max}>0$, we find numerically the values of $a_{0}$ and $b_{0}$ which minimize the Kullback-Leibler divergence between $G_{0}$ and a uniform distribution on the interval $\left[0,\lambda_{\max}\right]$. This Kullback-Leibler divergence can be computed explicitly as

$$-\log\lambda_{\max}-a_{0}\log b_{0}+\log\Gamma \left(a_{0}\right)-\left(a_{0}-1\right)\left(\log\lambda_{\max}-1\right)+\frac{b_{0}\lambda_{\max}}{2}.$$

In this new parameterization, our goal is to make a sensible choice for $\lambda_{\max}$. It is worth emphasizing that by this procedure we are not truncating the support of $G_{0}$, but only using the uniform distribution on the interval $\left[0,\lambda_{\max}\right]$ as a reference for our choice of the base measure hyperparameters $a_{0}$ and $b_{0}$.

Our proposal to choose $\lambda_{\max}$ goes as follows. We fix some value 0≤σ<1 for the discount parameter and choose an integer $k_{0}$ as the prior expectation of the number of clusters *K*, which, using the results at the end of Section 3, can be computed explicitly as

$$E\left[K\right]=\left\{\begin{array}{cc} \tau \times (\psi (\tau +T-p)-\psi (\tau )) & \mathrm{if}\;\sigma =0;\\ ({\left(\tau +\sigma \right)}_{T-p}/\left(\sigma \times {\left(\tau +1\right)}_{T-p-1}\right))-\tau /\sigma & \mathrm{if}\;\sigma >0, \end{array}\right.$$

in which ψ(x) is the digamma function (see [6] for a derivation of this result). Next, we find the value of the concentration parameter τ by solving $E\left[K\right]=k_{0}$ numerically. After this, for each $\lambda_{\max}$ in a grid of values, we run the Gibbs sampler and compute the posterior expectation of the number of clusters E[K∣y]. Finally, in the corresponding graph, we look for the value of $\lambda_{\max}$ located at the “elbow” of the curve, that is, the value of $\lambda_{\max}$ at which the values of E[K∣y] level off.

## 6. Simulated Data

**Figure 1:**
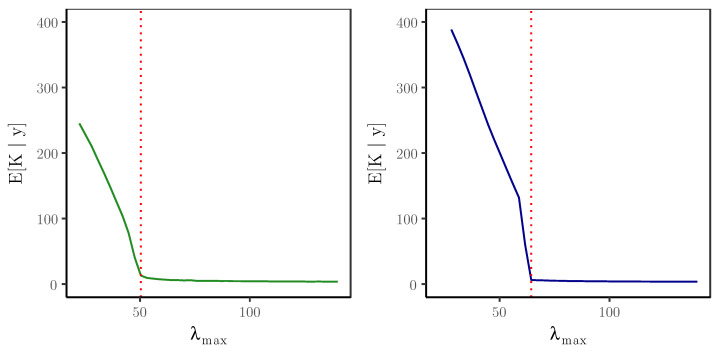
Formation of the elbows for σ=0.5 (left) and σ=0.75 (right). The red dotted lines indicate the chosen values of $\lambda_{\max}$.

As an explicit example of the graphical criterion in action, we used the functional form of a first-order model with thinning parameter α=0.15 to simulate a time series of length T=1000, for which the distribution of the innovations is a symmetric mixture of three Poisson distributions with parameters 1, 8, and 15. Figure 1 shows the formations of the elbows for two values of the discount parameter: σ=0.5 and σ=0.75.

**Figure 2:**
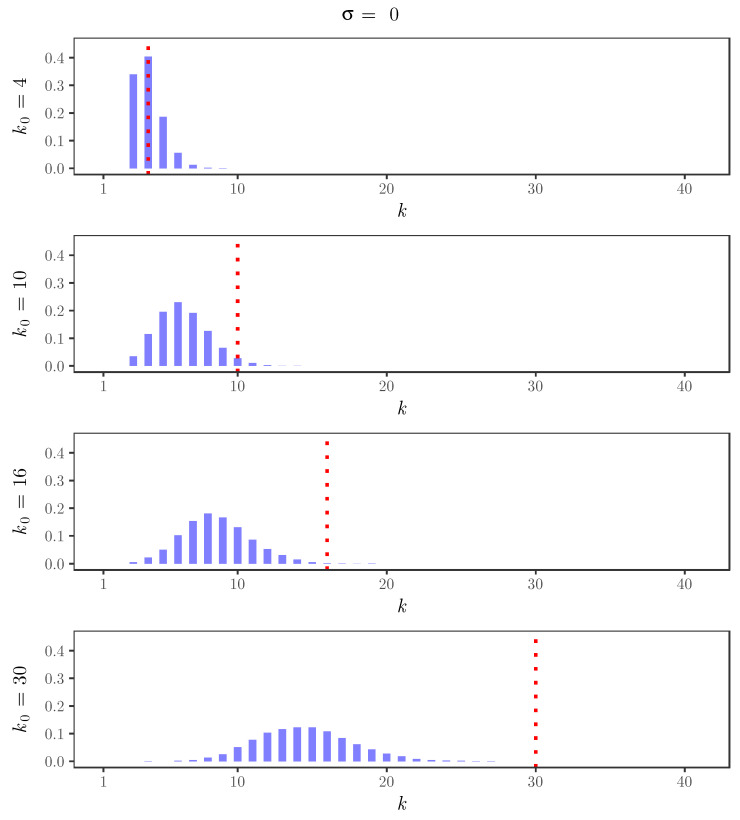
Posterior distributions of the number of clusters *K* for the simulated time series with σ=0 and $k_{0}=4,10,16,30$. The red dotted lines indicate the value of $k_{0}$.

**Figure 3:**
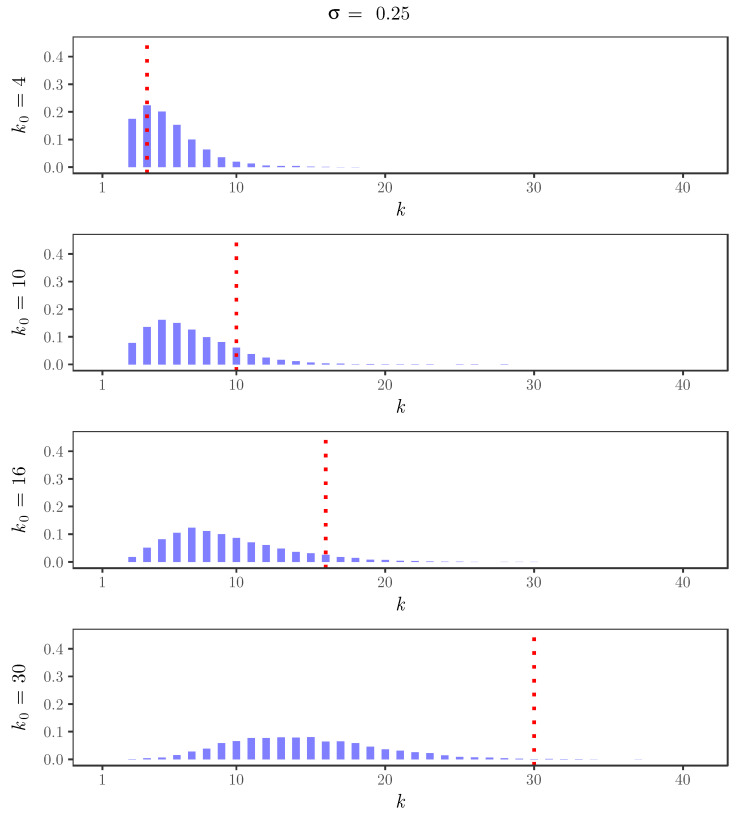
Posterior distributions of the number of clusters *K* for the simulated time series with σ=0.25 and $k_{0}=4,10,16,30$. The red dotted lines indicate the value of $k_{0}$.

**Figure 4:**
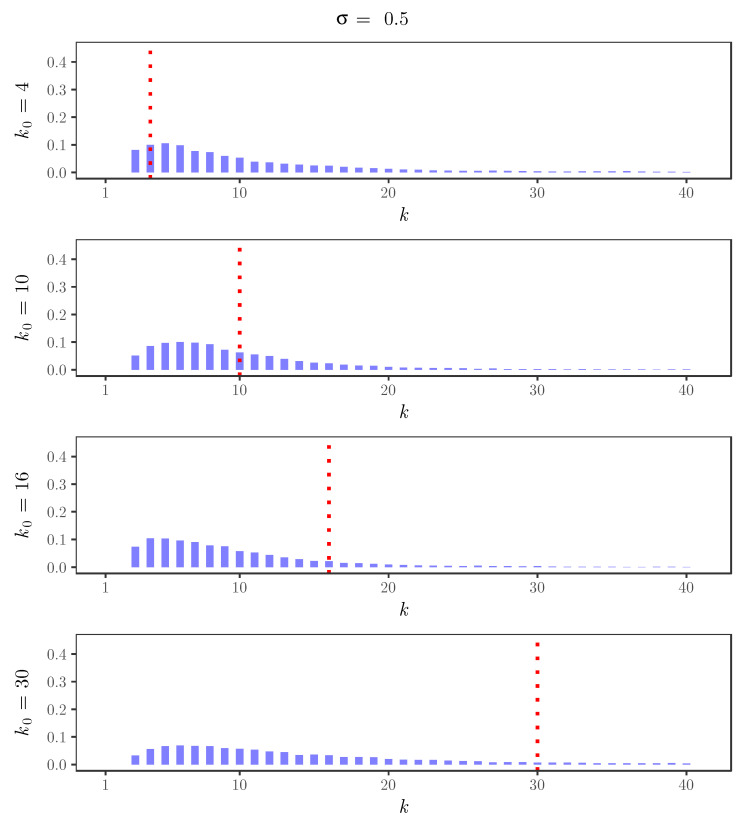
Posterior distributions of the number of clusters *K* for the simulated time series with σ=0.5 and $k_{0}=4,10,16,30$. The red dotted lines indicate the value of $k_{0}$.

**Figure 5:**
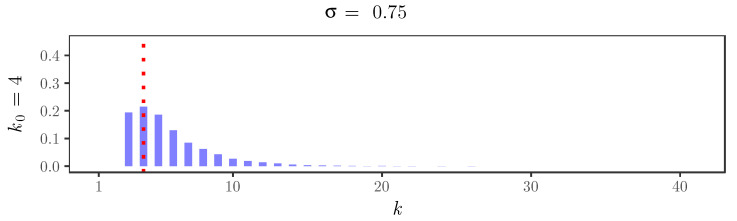

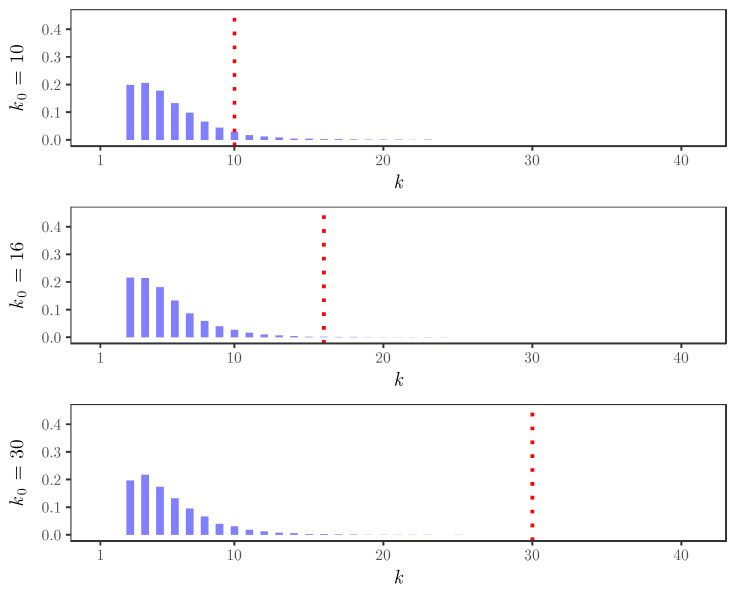
Posterior distributions of the number of clusters *K* for the simulated time series with σ=0.75 and $k_{0}=4,10,16,30$. The red dotted lines indicate the value of $k_{0}$.

For the simulated time series, Figure 2, Figure 3, Figure 4 and Figure 5 display the behavior of the posterior distributions obtained using the elbow method for $\left(k_{0},\sigma \right)\in \left\{4,10,16,30\right\}\times \left\{0,0.25,0.5,0.75\right\}$. These figures make the relation between the choice of the value of the discount parameter σ and the achieved robustness of the posterior distribution quite explicit: as we increase the value of the discount parameter σ, the posterior becomes insensitive to the choice of $k_{0}$. In particular, for σ=0.75, the posterior mode is always near 3, which is the number of components used in the distribution of the innovations of the simulated time series.

Once we understand the influence of the prior parameters on the robustness of the posterior distribution, an interesting question is how to get a point estimate for the distribution of clusters, in the sense that each $\lambda_{t}$, for t=p+1,⋯,T, would be assigned to one of the available clusters.

From the Gibbs sampler, we can easily get a Monte Carlo approximation for the probabilities $d_{rt}=\mathrm{Pr}\left\{\lambda_{r}\neq \lambda_{t}|y\right\}$, for r,t=p+1,⋯,T. These probabilities define a dissimilarity matrix $D=(d_{rt})$ among the innovation rates. Although *D* is not a distance matrix, we can use it as a starting point to represent the innovation rates in a two-dimensional Euclidean space using the technique of metric multidimensional scaling (see [18] for a general discussion). From this two-dimensional representation, we use hierarchical clustering techniques to build a dendrogram, which is appropriately cut in order to define three clusters, allowing us to assign a single cluster label to each innovation rate.

Table 1 displays the confusion matrix of this assignment, showing that 83% of the innovations were grouped correctly in the clusters which correspond to the mixture components used to simulate the time series.

## 7. Earthquake Data

In this section, we analyze a time series of yearly worldwide earthquakes events of substantial magnitude (equal or greater than 7 points on the Richter scale) from 1900 to 2018 (http://www.usgs.gov/natural-hazards/earthquake-hazards/earthquakes).

The forecasting performances of the INAR(*p*) and the PY-INAR(*p*) models are compared using a cross-validation procedure in which the models are trained with data ranging from the beginning of the time series up to a certain time, and predictions are made for epochs outside this training range.

Using this cross-validation procedure, we trained the INAR(*p*) and the PY-INAR(*p*) models with orders p=1,2,and3, and made one-step-ahead predictions. Table 2 shows the out-of-sample mean absolute errors (MAE) for the INAR(*p*) and the PY-INAR(*p*) models. In this table, the MAE’s are computed predicting the counts for the last 36 months. For the three model orders, the PY-INAR(*p*) model yields a smaller MAE than the original INAR(*p*) model.

## Figures and Tables

**Table 1:** Confusion matrix for the cluster assignments.

	True		
Predicted	1	2	3
1	297	32	0
2	11	217	42
3	0	84	316

**Table 2:** Out-of-sample MAE’s for the INAR(*p*) and the PY-INAR(*p*) models, with orders p=1,2,and3. The last column shows the relative variations of the MAE’s for the PY-INAR(*p*) models with respect to the corresponding MAE’s for the INAR(*p*) models.

	INAR	PY-INAR	$\Delta_{\mathrm{PY}\text{-}\mathrm{INAR}}$
p=1	3.861	3.583	−0.072
p=2	3.583	3.417	−0.046
p=3	3.972	3.305	−0.202
